# Why do we need international standards on responsible research publication for authors and editors?

**DOI:** 10.7189/jogh.03.020301

**Published:** 2013-12

**Authors:** Elizabeth Wager, Sabine Kleinert

**Affiliations:** 1 Sideview and University of Split School of Medicine, Split, Croatia; 2 *TheLancet*, London, UK

Peer-reviewed publication is a vital step in the research process and permits research findings to be communicated effectively to readers. The peer review process is designed to select work of relevance to particular audiences, to improve the quality of reporting, and thus increase its transparency (eg, allowing methods to be replicated) [1]. Although it is by no means perfect, there is some evidence that peer review performs these functions, or at least that the quality of articles tends to improve from submission to publication [2,3]. However, peer review cannot, by itself, prevent fraud or misconduct, although in some cases it may help detect them. The publication process is therefore based on a degree of trust in the honesty and intentions of authors, reviewers, and editors.

However, responsible conduct in publishing research is not always fully understood, and while egregious behaviour (such as copying a published article into a new document and submitting it to another journal with new author names) is easily recognised as misconduct (in this case, plagiarism), other practices may be harder to classify. Without a good understanding of the ethics and conventions of publication, it is possible for authors and editors to unwittingly overstep the mark and do something that others find unacceptable [4]. It is therefore helpful for journals and institutions to provide clear guidance for authors and editors on what is expected of them.

**Figure:**
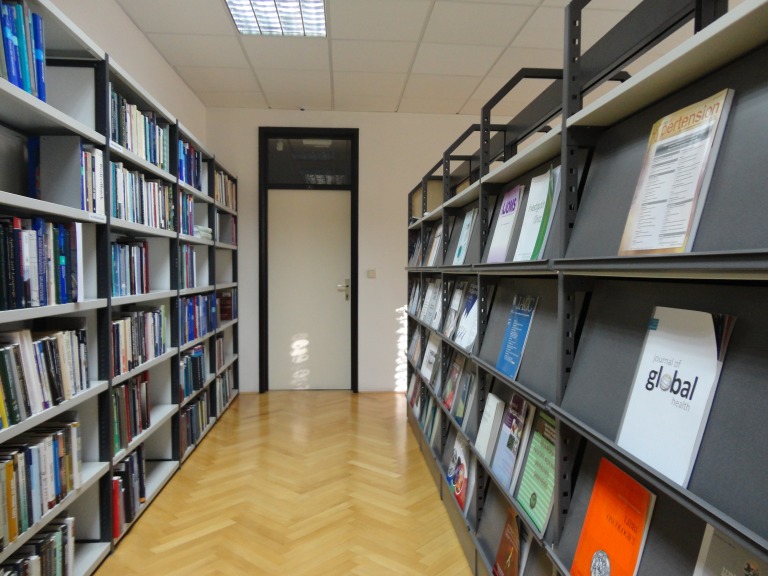
Photo: Courtesy of Leo Roglić, personal collection

Establishing clear expectations is particularly important for projects involving global or inter-disciplinary collaboration since authors’ experiences and publishing conventions may vary between countries, cultures, and disciplines [5]. For example, automatically adding the head of department’s name to a publication may be viewed as an expected courtesy in some regions, but as an unacceptable form of guest authorship in others.

This pair of guidelines, one for authors (Box 1, Table 1), the other for editors (Box 2, Table 2), was designed to emphasize that all parties have responsibilities. Many journal guidelines focus on unacceptable behaviour by authors with little or no recognition that editors may abuse their powers and have important accountabilities. Once again, the peer review process depends on trust. Authors from small institutions or low-income countries may be concerned that editors or reviewers may be prejudiced against their work; therefore these guidelines are aimed to promote fairness in peer review. (Guidelines for peer reviewers have also been developed by the Committee on Publication Ethics and these are another useful resource [6]).

Box 1 **Summary – Responsible research publication: International standards for authors. A position statement developed at the 2^nd^ World Conference on Research Integrity, Singapore, July 22-24, 2010**

• The research being reported should have been conducted in an ethical and responsible manner and should comply with all relevant legislation.

• Researchers should present their results clearly, honestly, and without fabrication, falsification or inappropriate data manipulation.

• Researchers should strive to describe their methods clearly and unambiguously so that their findings can be confirmed by others.

• Researchers should adhere to publication requirements that submitted work is original, is not plagiarised, and has not been published elsewhere.

• Authors should take collective responsibility for submitted and published work.

•The authorship of research publications should accurately reflect individuals’ contributions to the work and its reporting.

• Funding sources and relevant conflicts of interest should be disclosed.

**Table 1:** Responsible research publication: International standards for **authors**

**Introduction** Publication is the final stage of research and therefore a responsibility for all researchers. Scholarly publications are expected to provide a detailed and permanent record of research. Because publications form the basis for both new research and the application of findings, they can affect not only the research community but also, indirectly, society at large. Researchers therefore have a responsibility to ensure that their publications are honest, clear, accurate, complete and balanced, and should avoid misleading, selective or ambiguous reporting. Journal editors also have responsibilities for ensuring the integrity of the research literature and these are set out in companion guidelines. This document aims to establish international standards for authors of scholarly research publications and to describe responsible research reporting practice. We hope these standards will be endorsed by research institutions, funders, and professional societies; promoted by editors and publishers; and will aid in research integrity training.	
**1. Soundness and reliability**	
**1.1**	The research being reported should have been conducted in an ethical and responsible manner and follow all relevant legislation. *[See also the Singapore Statement on Research Integrity, www.singaporestatement.org]*
**1.2**	The research being reported should be sound and carefully executed.
**1.3**	Researchers should use appropriate methods of data analysis and display (and, if needed, seek and follow specialist advice on this).
**1.4**	Authors should take collective responsibility for their work and for the content of their publications. Researchers should check their publications carefully at all stages to ensure methods and findings are reported accurately. Authors should carefully check calculations, data presentations, typescripts/submissions and proofs.
**2. Honesty**	
**2.1**	Researchers should present their results honestly and without fabrication, falsification or inappropriate data manipulation. Research images (eg, micrographs, x-rays, pictures of electrophoresis gels) should not be modified in a misleading way.
**2.2**	Researchers should strive to describe their methods and to present their findings clearly and unambiguously. Researchers should follow applicable reporting guidelines. Publications should provide sufficient detail to permit experiments to be repeated by other researchers.
**2.3**	Reports of research should be complete. They should not omit inconvenient, inconsistent or inexplicable findings or results that do not support the authors’ or sponsors’ hypothesis or interpretation.
**2.4**	Research funders and sponsors should not be able to veto publication of findings that do not favour their product or position. Researchers should not enter agreements that permit the research sponsor to veto or control the publication of the findings (unless there are exceptional circumstances, such as research classified by governments because of security implications).
**2.5**	Authors should alert the editor promptly if they discover an error in any submitted, accepted or published work. Authors should cooperate with editors in issuing corrections or retractions when required.
**2.6**	Authors should represent the work of others accurately in citations and quotations.
**2.7**	Authors should not copy references from other publications if they have not read the cited work.
**3. Balance**	
**3.1**	New findings should be presented in the context of previous research. The work of others should be fairly represented. Scholarly reviews and syntheses of existing research should be complete, balanced, and should include findings regardless of whether they support the hypothesis or interpretation being proposed. Editorials or opinion pieces presenting a single viewpoint or argument should be clearly distinguished from scholarly reviews.
**3.2**	Study limitations should be addressed in publications.
**4. Originality**	
**4.1**	Authors should adhere to publication requirements that submitted work is original and has not been published elsewhere in any language. Work should not be submitted concurrently to more than one publication unless the editors have agreed to co-publication. If articles are co-published this fact should be made clear to readers.
**4.2**	Applicable copyright laws and conventions should be followed. Copyright material (eg, tables, figures or extensive quotations) should be reproduced only with appropriate permission and acknowledgement.
**4.3**	Relevant previous work and publications, both by other researchers and the authors’ own, should be properly acknowledged and referenced. The primary literature should be cited where possible.
**4.4**	Data, text, figures or ideas originated by other researchers should be properly acknowledged and should not be presented as if they were the authors’ own. Original wording taken directly from publications by other researchers should appear in quotation marks with the appropriate citations.
**4.5**	Authors should inform editors if findings have been published previously or if multiple reports or multiple analyses of a single data set are under consideration for publication elsewhere. Authors should provide copies of related publications or work submitted to other journals.
**4.6**	Multiple publications arising from a single research project should be clearly identified as such and the primary publication should be referenced. Translations and adaptations for different audiences should be clearly identified as such, should acknowledge the original source, and should respect relevant copyright conventions and permission requirements. If in doubt, authors should seek permission from the original publisher before republishing any work.
**5. Transparency**	
**5.1**	All sources of research funding, including direct and indirect financial support, supply of equipment or materials, and other support (such as specialist statistical or writing assistance) should be disclosed.
**5.2**	Authors should disclose the role of the research funder(s) or sponsor (if any) in the research design, execution, analysis, interpretation and reporting.
**5.3**	Authors should disclose relevant financial and non-financial interests and relationships that might be considered likely to affect the interpretation of their findings or which editors, reviewers or readers might reasonably wish to know. This includes any relationship to the journal, for example if editors publish their own research in their own journal. In addition, authors should follow journal and institutional requirements for disclosing competing interests.
**6. Appropriate authorship and acknowledgement**	
**6.1**	The research literature serves as a record not only of what has been discovered but also of who made the discovery. The authorship of research publications should therefore accurately reflect individuals’ contributions to the work and its reporting.
**6.2**	In cases where major contributors are listed as authors while those who made less substantial, or purely technical, contributions to the research or to the publication are listed in an acknowledgement section, the criteria for authorship and acknowledgement should be agreed at the start of the project. Ideally, authorship criteria within a particular field should be agreed, published and consistently applied by research institutions, professional and academic societies, and funders. While journal editors should publish and promote accepted authorship criteria appropriate to their field, they cannot be expected to adjudicate in authorship disputes. Responsibility for the correct attribution of authorship lies with authors themselves working under the guidance of their institution. Research institutions should promote and uphold fair and accepted standards of authorship and acknowledgement. When required, institutions should adjudicate in authorship disputes and should ensure that due process is followed.
**6.3**	Researchers should ensure that only those individuals who meet authorship criteria (ie, made a substantial contribution to the work) are rewarded with authorship and that deserving authors are not omitted. Institutions and journal editors should encourage practices that prevent guest, gift, and ghost authorship. Note: • guest authors are those who do not meet accepted authorship criteria but are listed because of their seniority, reputation or supposed influence • gift authors are those who do not meet accepted authorship criteria but are listed as a personal favour or in return for payment • ghost authors are those who meet authorship criteria but are not listed
**6.4**	All authors should agree to be listed and should approve the submitted and accepted versions of the publication. Any change to the author list should be approved by all authors including any who have been removed from the list. The corresponding author should act as a point of contact between the editor and the other authors and should keep co-authors informed and involve them in major decisions about the publication (eg, responding to reviewers’ comments).
**6.5**	Authors should not use acknowledgements misleadingly to imply a contribution or endorsement by individuals who have not, in fact, been involved with the work or given an endorsement.
**7. Accountability and responsibility**	
**7.1**	All authors should have read and be familiar with the reported work and should ensure that publications follow the principles set out in these guidelines. In most cases, authors will be expected to take joint responsibility for the integrity of the research and its reporting. However, if authors take responsibility only for certain aspects of the research and its reporting, this should be specified in the publication.
**7.2**	Authors should work with the editor or publisher to correct their work promptly if errors or omissions are discovered after publication.
**7.3**	Authors should abide by relevant conventions, requirements, and regulations to make materials, reagents, software or data sets available to other researchers who request them. Researchers, institutions, and funders should have clear policies for handling such requests. Authors must also follow relevant journal standards. While proper acknowledgement is expected, researchers should not demand authorship as a condition for sharing materials.
**7.4**	Authors should respond appropriately to post-publication comments and published correspondence. They should attempt to answer correspondents’ questions and supply clarification or additional details where needed.
**8. Adherence to peer review and publication conventions**	
**8.1**	Authors should follow publishers’ requirements that work is not submitted to more than one publication for consideration at the same time.
**8.2**	Authors should inform the editor if they withdraw their work from review, or choose not to respond to reviewer comments after receiving a conditional acceptance.
**8.3**	Authors should respond to reviewers’ comments in a professional and timely manner.
**8.4**	Authors should respect publishers’ requests for press embargos and should not generally allow their findings to be reported in the press if they have been accepted for publication (but not yet published) in a scholarly publication. Authors and their institutions should liaise and cooperate with publishers to coordinate media activity (eg, press releases and press conferences) around publication. Press releases should accurately reflect the work and should not include statements that go further than the research findings.
**9. Responsible reporting of research involving humans or animals**	
**9.1**	Appropriate approval, licensing or registration should be obtained before the research begins and details should be provided in the report (eg, Institutional Review Board, Research Ethics Committee approval, national licensing authorities for the use of animals).
**9.2**	If requested by editors, authors should supply evidence that reported research received the appropriate approval and was carried out ethically (eg, copies of approvals, licences, participant consent forms).
**9.3**	Researchers should not generally publish or share identifiable individual data collected in the course of research without specific consent from the individual (or their representative). Researchers should remember that many scholarly journals are now freely available on the internet, and should therefore be mindful of the risk of causing danger or upset to unintended readers (eg, research participants or their families who recognise themselves from case studies, descriptions, images or pedigrees).
**9.4**	The appropriate statistical analyses should be determined at the start of the study and a data analysis plan for the prespecified outcomes should be prepared and followed. Secondary or *post hoc* analyses should be distinguished from primary analyses and those set out in the data analysis plan.
**9.5**	Researchers should publish all meaningful research results that might contribute to understanding. In particular, there is an ethical responsibility to publish the findings of all clinical trials. The publication of unsuccessful studies or experiments that reject a hypothesis may help prevent others from wasting time and resources on similar projects. If findings from small studies and those that fail to reach statistically significant results can be combined to produce more useful information (eg, by meta-analysis) then such findings should be published.
**9.6**	Authors should supply research protocols to journal editors if requested (eg, for clinical trials) so that reviewers and editors can compare the research report to the protocol to check that it was carried out as planned and that no relevant details have been omitted. Researchers should follow relevant requirements for clinical trial registration and should include the trial registration number in all publications arising from the trial.

Box 2 **Summary – Responsible research publication: International standards for editors. A position statement developed at the 2^nd^ World Conference on Research Integrity, Singapore, July 22-24, 2010**

• Editors are accountable and should take responsibility for everything they publish.

• Editors should make fair and unbiased decisions independent from commercial consideration and ensure a fair and appropriate peer review process.

• Editors should adopt editorial policies that encourage maximum transparency and complete, honest reporting.

• Editors should guard the integrity of the published record by issuing corrections and retractions when needed and pursuing suspected or alleged research and publication misconduct.

• Editors should pursue reviewer and editorial misconduct.

• Editors should critically assess the ethical conduct of studies in humans and animals.

• Peer reviewers and authors should be told what is expected of them.

• Editors should have appropriate policies in place for handling editorial conflicts of interest.

**Table 2:** Responsible research publication: International standards for **editors**

**Introduction** As guardians and stewards of the research record, editors should encourage authors to strive for, and adhere themselves to, the highest standards of publication ethics. Furthermore, editors are in a unique position to indirectly foster responsible conduct of research through their policies and processes. To achieve the maximum effect within the research community, ideally all editors should adhere to universal standards and good practices. While there are important differences between different fields and not all areas covered are relevant to each research community, there are important common editorial policies, processes, and principles that editors should follow to ensure the integrity of the research record. These guidelines are a starting point and are aimed at journal editors in particular. While books and monographs are important and relevant research records in many fields, guidelines for book editors are beyond the scope of these recommendations. It is hoped that in due course such guidelines can be added to this document. Editors should regard themselves as part of the wider professional editorial community, keep themselves abreast of relevant policies and developments, and ensure their editorial staff is trained and kept informed of relevant issues. To be a good editor requires many more principles than are covered here. These suggested principles, policies, and processes are particularly aimed at fostering research and publication integrity.	
**Editorial Principles**	
**1. Accountability and responsibility for journal content** Editors have to take responsibility for everything they publish and should have procedures and policies in place to ensure the quality of the material they publish and maintain the integrity of the published record (see paragraphs 4-8).	
**2. Editorial independence and integrity** An important part of the responsibility to make fair and unbiased decisions is the upholding of the principle of editorial independence and integrity.	
**2.1**	**Separating decision-making from commercial considerations** Editors should make decisions on academic merit alone and take full responsibility for their decisions. Processes must be in place to separate commercial activities within a journal from editorial processes and decisions. Editors should take an active interest in the publisher’s pricing policies and strive for wide and affordable accessibility of the material they publish. Sponsored supplements must undergo the same rigorous quality control and peer review as any other content for the journal. Decisions on such material must be made in the same way as any other journal content. The sponsorship and role of the sponsor must be clearly declared to readers. Advertisements need to be checked so that they follow journal guidelines, should be clearly distinguishable from other content, and should not in any way be linked to scholarly content.
**2.2**	**Editors’ relationship to the journal publisher or owner** Editors should ideally have a written contract setting out the terms and conditions of their appointment with the journal publisher or owner. The principle of editorial independence should be clearly stated in this contract. Journal publishers and owners should not have any role in decisions on content for commercial or political reasons. Publishers should not dismiss an editor because of any journal content unless there was gross editorial misconduct or an independent investigation has concluded that the editor’s decision to publish was against the journal’s scholarly mission.
**2.3**	**Journal metrics and decision-making** Editors should not attempt to inappropriately influence their journal’s ranking by artificially increasing any journal metric. For example, it is inappropriate to demand that references to that journal’s articles are included except for genuine scholarly reasons. In general, editors should ensure that papers are reviewed on purely scholarly grounds and that authors are not pressured to cite specific publications for non-scholarly reasons.
**3. Editorial confidentiality**	
**3.1**	**Authors’ material** If a journal operates a system where peer reviewers are chosen by editors (rather than posting papers for all to comment as a pre-print version), editors must protect the confidentiality of authors’ material and remind reviewers to do so as well. In general, editors should not share submitted papers with editors of other journals, unless with the authors’ agreement or in cases of alleged misconduct (see below). Editors are generally under no obligation to provide material to lawyers for court cases. Editors should not give any indication of a paper’s status with the journal to anyone other than the authors. Web-based submission systems must be run in a way that prevents unauthorised access. In the case of a misconduct investigation, it may be necessary to disclose material to third parties (eg, an institutional investigation committee or other editors).
**3.2**	**Reviewers** Editors should protect reviewers’ identities unless operating an open peer review system. However, if reviewers wish to disclose their names, this should be permitted. If there is alleged or suspected reviewer misconduct it may be necessary to disclose a reviewer’s name to a third party.
**General Editorial Policies**	
**4. Encourage maximum transparency and complete and honest reporting** To advance knowledge in scholarly fields, it is important to understand why particular work was done, how it was planned and conducted and by whom, and what it adds to current knowledge. To achieve this understanding, maximum transparency and complete and honest reporting are crucial.	
**4.1**	**Authorship and responsibility** Journals should have a clear policy on authorship that follows the standards within the relevant field. They should give guidance in their information for authors on what is expected of an author and, if there are different authorship conventions within a field, they should state which they adhere to. For multidisciplinary and collaborative research, it should be apparent to readers who has done what and who takes responsibility for the conduct and validity of which aspect of the research. Each part of the work should have at least one author who takes responsibility for its validity. For example, individual contributions and responsibilities could be stated in a contributor section. All authors are expected to have contributed significantly to the paper and to be familiar with its entire content and ideally, this should be declared in an authorship statement submitted to the journal. When there are undisputed changes in authorship for appropriate reasons, editors should require that all authors (including any whose names are being removed from an author list) agree these in writing. Authorship disputes (ie, disagreements on who should or should not be an author before or after publication) cannot be adjudicated by editors and should be resolved at institutional level or through other appropriate independent bodies for both published and unpublished papers. Editors should then act on the findings, for example by correcting authorship in published papers. Journals should have a publicly declared policy on how papers submitted by editors or editorial board members are handled (see paragraph on editorial conflicts of interest: 8.2)
**4.2**	**Conflicts of interest and role of the funding source** Editors should have policies that require all authors to declare any relevant financial and non-financial conflicts of interest and publish at least those that might influence a reader’s perception of a paper, alongside the paper. The funding source of the research should be declared and published, and the role of the funding source in the conception, conduct, analysis, and reporting of the research should be stated and published. Editors should make it clear in their information for authors if in certain sections of the journal (eg, commissioned commentaries or review articles) certain conflicts of interest preclude authorship.
**4.3**	**Full and honest reporting and adherence to reporting guidelines** Among the most important responsibilities of editors is to maintain a high standard in the scholarly literature. Although standards differ among journals, editors should work to ensure that all published papers make a substantial new contribution to their field. Editors should discourage so-called ‘salami publications’ (ie, publication of the minimum publishable unit of research), avoid duplicate or redundant publication unless it is fully declared and acceptable to all (eg, publication in a different language with cross-referencing), and encourage authors to place their work in the context of previous work (ie, to state why this work was necessary/done, what this work adds or why a replication of previous work was required, and what readers should take away from it). Journals should adopt policies that encourage full and honest reporting, for example, by requiring authors in fields where it is standard to submit protocols or study plans, and, where they exist, to provide evidence of adherence to relevant reporting guidelines. Although devised to improve reporting, adherence to reporting guidelines also makes it easier for editors, reviewers, and readers to judge the actual conduct of the research. Digital image files, figures, and tables should adhere to the appropriate standards in the field. Images should not be inappropriately altered from the original or present findings in a misleading way. Editors might also consider screening for plagiarism, duplicate or redundant publication by using anti-plagiarism software, or for image manipulation. If plagiarism or fraudulent image manipulation is detected, this should be pursued with the authors and relevant institutions (see paragraph on how to handle misconduct: 5.2
**5. Responding to criticisms and concerns** Reaction and response to published research by other researchers is an important part of scholarly debate in most fields and should generally be encouraged. In some fields, journals can facilitate this debate by publishing readers’ responses. Criticisms may be part of a general scholarly debate but can also highlight transgressions of research or publication integrity.	
**5.1**	**Ensuring integrity of the published record - corrections** When genuine errors in published work are pointed out by readers, authors, or editors, which do not render the work invalid, a correction (or erratum) should be published as soon as possible. The online version of the paper may be corrected with a date of correction and a link to the printed erratum. If the error renders the work or substantial parts of it invalid, the paper should be retracted with an explanation as to the reason for retraction (ie, honest error).
**5.2**	**Ensuring the integrity of the published record – suspected research or publication misconduct** If serious concerns are raised by readers, reviewers, or others, about the conduct, validity, or reporting of academic work, editors should initially contact the authors (ideally all authors) and allow them to respond to the concerns. If that response is unsatisfactory, editors should take this to the institutional level (see below). In rare cases, mostly in the biomedical field, when concerns are very serious and the published work is likely to influence clinical practice or public health, editors should consider informing readers about these concerns, for example by issuing an ‘expression of concern’, while the investigation is ongoing. Once an investigation is concluded, the appropriate action needs to be taken by editors with an accompanying comment that explains the findings of the investigation. Editors should also respond to findings from national research integrity organisations that indicate misconduct relating to a paper published in their journal. Editors can themselves decide to retract a paper if they are convinced that serious misconduct has happened even if an investigation by an institution or national body does not recommend it. Editors should respond to all allegations or suspicions of research or publication misconduct raised by readers, reviewers, or other editors. Editors are often the first recipients of information about such concerns and should act, even in the case of a paper that has not been accepted or has already been rejected. Beyond the specific responsibility for their journal’s publications, editors have a collective responsibility for the research record and should act whenever they become aware of potential misconduct if at all possible. Cases of possible plagiarism or duplicate/redundant publication can be assessed by editors themselves. However, in most other cases, editors should request an investigation by the institution or other appropriate bodies (after seeking an explanation from the authors first and if that explanation is unsatisfactory). Retracted papers should be retained online, and they should be prominently marked as a retraction in all online versions, including the PDF, for the benefit of future readers. For further guidance on specific allegations and suggested actions, such as retractions, see the COPE flowcharts and retraction guidelines (http://publicationethics.org/flowcharts; http://publicationethics.org/files/u661/Retractions_COPE_gline_final_3_Sept_09__2_.pdf).
**5.3**	**Encourage scholarly debate** All journals should consider the best mechanism by which readers can discuss papers, voice criticisms, and add to the debate (in many fields this is done via a print or online correspondence section). Authors may contribute to the debate by being allowed to respond to comments and criticisms where relevant. Such scholarly debate about published work should happen in a timely manner. Editors should clearly distinguish between criticisms of the limitations of a study and criticisms that raise the possibility of research misconduct. Any criticisms that raise the possibility of misconduct should not just be published but should be further investigated even if they are received a long time after publication.
**Editorial Policies Relevant only to Journals that Publish Research in Humans or Animals**	
**6. Critically assess and require a high standard of ethical conduct of research** Especially in biomedical research but also in social sciences and humanities, ethical conduct of research is paramount in the protection of humans and animals. Ethical oversight, appropriate consent procedures, and adherence to relevant laws are required from authors. Editors need to be vigilant to concerns in this area.	
**6.1**	**Ethics approval and ethical conduct** Editors should generally require approval of a study by an ethics committee (or institutional review board) and the assurance that it was conducted according to the Declaration of Helsinki for medical research in humans but, in addition, should be alert to areas of concern in the ethical conduct of research. This may mean that a paper is sent to peer reviewers with particular expertise in this area, to the journal’s ethics committee if there is one, or that editors require further reassurances or evidence from authors or their institutions. Papers may be rejected on ethical grounds even if the research had ethics committee approval.
**6.2**	**Consent (to take part in research)** If research is done in humans, editors should ensure that a statement on the consent procedure is included in the paper. In most cases, written informed consent is the required norm. If there is any concern about the consent procedure, if the research is done in vulnerable groups, or if there are doubts about the ethical conduct, editors should ask to see the consent form and enquire further from authors, exactly how consent was obtained.
**6.3**	**Consent (for publication)** For all case reports, small case series, and images of people, editors should require the authors to have obtained explicit consent for publication (which is different from consent to take part in research). This consent should inform participants which journal the work will be published in, make it clear that, although all efforts will be made to remove unnecessary identifiers, complete anonymity is not possible, and ideally state that the person described has seen and agreed with the submitted paper. The signed consent form should be kept with the patient file rather than sent to the journal (to maximise data protection and confidentiality, see paragraph 6.4). There may be exceptions where it is not possible to obtain consent, for example when the person has died. In such cases, a careful consideration about possible harm is needed and out of courtesy attempts should be made to obtain assent from relatives. In very rare cases, an important public health message may justify publication without consent if it is not possible despite all efforts to obtain consent and the benefit of publication outweighs the possible harm.
**6.4**	**Data protection and confidentiality** Editors should critically assess any potential breaches of data protection and patient confidentiality. This includes requiring properly informed consent for the actual research presented, consent for publication where applicable (see paragraph 6.3), and having editorial policies that comply with guidelines on patient confidentiality.
**6.5**	**Adherence to relevant laws and best practice guidelines for ethical conduct** Editors should require authors to adhere to relevant national and international laws and best practice guidelines where applicable, for example when undertaking animal research. Editors should encourage registration of clinical trials.
**Editorial Processes**	
**7. Ensuring a fair and appropriate peer review process** One of the most important responsibilities of editors is organising and using peer review fairly and wisely. Editors should explain their peer review processes in the information for authors and also indicate which parts of the journal are peer reviewed.	
**7.1**	**Decision whether to review** Editors may reject a paper without peer review when it is deemed unsuitable for the journal’s readers or is of poor quality. This decision should be made in a fair and unbiased way. The criteria used to make this decision should be made explicit. The decision not to send a paper for peer review should only be based on the academic content of the paper, and should not be influenced by the nature of the authors or the host institution.
**7.2**	**Interaction with peer reviewers** Editors should use appropriate peer reviewers for papers that are considered for publication by selecting people with sufficient expertise and avoiding those with conflicts of interest. Editors should ensure that reviews are received in a timely manner. Peer reviewers should be told what is expected of them and should be informed about any changes in editorial policies. In particular, peer reviewers should be asked to assess research and publication ethics issues (ie, whether they think the research was done and reported ethically, or if they have any suspicions of plagiarism, fabrication, falsification, or redundant publication). Editors should have a policy to request a formal conflict of interest declaration from peer reviewers and should ask peer reviewers to inform them about any such conflict of interest at the earliest opportunity so that they can make a decision on whether an unbiased review is possible. Certain conflicts of interest may disqualify a peer reviewer. Editors should stress confidentiality of the material to peer reviewers and should require peer reviewers to inform them when they ask a colleague for help with a review or if they mentor a more junior colleague in conducting peer review. Editors should ideally have a mechanism to monitor the quality and timeliness of peer review and to provide feedback to reviewers.
**7.3**	**Reviewer misconduct** Editors must take reviewer misconduct seriously and pursue any allegation of breach of confidentiality, non-declaration of conflicts of interest (financial or non-financial), inappropriate use of confidential material, or delay of peer review for competitive advantage. Allegations of serious reviewer misconduct, such as plagiarism, should be taken to the institutional level (for further guidance see: http://publicationethics.org/files/u2/07_Reviewer_misconduct.pdf).
**7.4**	**Interaction with authors** Editors should make it clear to authors what the role of the peer reviewer is because this may vary from journal to journal. Some editors regard peer reviewers as advisors and may not necessarily follow (or even ask for) reviewers’ recommendations on acceptance or rejection. Correspondence from editors is usually with the corresponding author, who should guarantee to involve co-authors at all stages. Communicating with all authors at first submission and at final acceptance stage can be helpful to ensure all authors are aware of the submission and have approved the publication. Normally, editors should pass on all peer reviewers’ comments in their entirety. However, in exceptional cases, it may be necessary to exclude parts of a review, if it, for example, contains libellous or offensive remarks. It is important, however, that such editorial discretion is not inappropriately used to suppress inconvenient comments. There should always be good reasons, which are clearly communicated to authors, if additional reviewers are sought at a late stage in the process. The final editorial decision and reasons for this should be clearly communicated to authors and reviewers. If a paper is rejected, editors should ideally have an appeals process. Editors, however, are not obliged to overturn their decision.
**8. Editorial decision-making** Editors are in a powerful position by making decisions on publications, which makes it very important that this process is as fair and unbiased as possible, and is in accordance with the academic vision of the particular journal.	
**8.1**	**Editorial and journal processes** All editorial processes should be made clear in the information for authors. In particular, it should be stated what is expected of authors, which types of papers are published, and how papers are handled by the journal. All editors should be fully familiar with the journal policies, vision, and scope. The final responsibility for all decisions rests with the editor-in-chief.
**8.2**	**Editorial conflicts of interest** Editors should not be involved in decisions about papers in which they have a conflict of interest, for example if they work or have worked in the same institution and collaborated with the authors, if they own stock in a particular company, or if they have a personal relationship with the authors. Journals should have a defined process for handling such papers. Journals should also have a process in place to handle papers submitted by editors or editorial board members to ensure unbiased and independent handling of such papers. This process should be stated in the information for authors. Editorial conflicts of interests should be declared, ideally publicly.

The guidelines on responsible research reporting were developed after wide international consultation with input from almost all parts of the world (Box 3). We are delighted that they are being promoted by the *Journal of Global Health* and hope they will be taken up by other journals. One practical reason for developing the guidelines was to spare journals and institutions the work involved in developing their own guidelines from scratch and we are happy for them to be referenced or adapted as required.

Box 3 **Responsible research publication position statements: Background information and acknowledgements**

Note: This has been previously published in: Mayer T & Steneck N (eds) *Promoting Research Integrity in a Global Environment*. Imperial College Press / World Scientific Publishing, Singapore (Chapter 49, pp 305-7). ISBN 978-981-4340-97-7

The following position statements were developed at the 2^nd^ World Conference on Research Integrity, held in Singapore in July 2010. They are designed to complement the Singapore Statement and to provide more detailed guidance on responsible research publication with particular emphasis on research integrity and publication ethics. The first statement is aimed at researchers in their role as authors of publications. The second statement is aimed at editors of scholarly journals that publish research.

The two statements were originally drafted by the named authors (Elizabeth Wager and Sabine Kleinert, the Chair and Vice-Chair of the Committee on Publication Ethics – COPE). These drafts were circulated before the meeting, discussed with the invited speakers, and revised to reflect these discussions. At the meeting in Singapore, the revised draft documents were presented and discussed in two sessions and further refined during a one-day, post-conference workshop. Both statements were then reworked to reflect the discussions in Singapore and circulated to those who had participated in the sessions and to members of the COPE Council and the International Council for Science (ICSU). However, while we hope such organizations may endorse the statements, they are primarily based on the views of participants at the Singapore meeting and therefore do not necessarily represent the official views of any of the participating organizations or the individuals’ institutions.

While some differences in publishing conventions exist between fields, it was evident from the discussion that there is much common ground and also a desire to raise standards in the reporting of research. The two documents therefore aim to establish standards for authors and editors of scholarly research publications and to describe responsible research reporting and publishing practice. Given the special issues raised by research involving humans or animals, which may not apply to other types of research, both statements include a specific section on these. We hope the statements will be endorsed by research institutions, funders, professional societies, and publishers.

While it would be impossible to reflect the views of all researchers and editors, we were pleased to involve participants and reviewers from a wide range of academic fields including biology, forestry, earth sciences, the humanities, mathematics, medicine, philosophy, and political science. The Singapore meeting also brought together participants from Africa, Asia, Australasia, Europe, the Middle East, and North America. We hope the versions presented here reflect the lively debate that took place before, during and after the meeting. However, we also hope that participants and reviewers will appreciate that it was not possible to incorporate all the suggestions we received, because some were contradictory. We therefore offer these documents as the first step in a process aimed at improving the reporting and publication of research and hope the statements will be reviewed and revised, as necessary, at future meetings.

We thank the following people who contributed to the discussions in Singapore and commented on drafts:

Siti Akmar Abu Samah (Universiti Teknologi MARA, Malaysia), Riaz Agha (*International Journal of Surgery*), Douglas Arnold (University of Minnesota and Society for Industrial and Applied Mathematics), Virginia Barbour (*Public Library of Science, PLoS Medicine*), Trish Groves (*BMJ*), Sara Jordan (Department of Politics & Public Administration, University of Hong Kong), Kamaruzaman Jusoff (Faculty of Forestry, Universiti Putra Malaysia), Abdellatif Maamri (Training Institute for Health Careers, Health Ministry, Oujda, Morocco), Ben Martin (*Research Policy*), Ana Marušić (*Croatian Medical Journal*), Linda Miller (*Nature,* now at New York University School of Medicine), Syntia Nchangwi (Cameroon), BJC Perera (*Sri Lanka Journal of Child Health, Sri Lanka Journal of Bio-Medical Informatics, Ceylon Medical Journal),* Bernd Pulverer (European Molecular Biology Organization), Margaret Rees (*Maturitas*), Iveta Simera (EQUATOR Network), Randell Stephenson (*Journal of Geodynamics*), Xiongyong Sun (China National Knowledge Infrastructure), Diane Sullenberger (*Proceedings of the National Academy of Sciences*), David Vaux (La Trobe University, Australia), Vasiliy Vlassov (Society for Evidence Based Medicine, Moscow, Russia).

Developments in global health require the effective communication of research findings so they can contribute to a useful and reliable evidence-base that readers can trust. We hope that the statements are helpful for readers, authors, and editors.
